# HLA supertype variation across populations: new insights into the role of natural selection in the evolution of HLA-A and HLA-B polymorphisms

**DOI:** 10.1007/s00251-015-0875-9

**Published:** 2015-10-12

**Authors:** Rodrigo dos Santos Francisco, Stéphane Buhler, José Manuel Nunes, Bárbara Domingues Bitarello, Gustavo Starvaggi França, Diogo Meyer, Alicia Sanchez-Mazas

**Affiliations:** Department of Genetics and Evolutionary Biology, University of São Paulo, São Paulo, Brazil; Laboratory of Anthropology, Genetics and Peopling History, Department of Genetics and Evolution–Anthropology Unit, University of Geneva, Geneva, Switzerland; Hospital Israelita Albert Einstein, São Paulo, Brazil; Transplantation Immunology Unit and National Reference Laboratory for Histocompatibility, Department of Genetic and Laboratory Medicine, Geneva University Hospital, Geneva, Switzerland; Institute of Genetics and Genomics in Geneva (IGE3), Geneva, Switzerland; Department of Biochemistry, Chemistry Institute, University of São Paulo, São Paulo, Brazil; Molecular Oncology Center, Sírio-Libanês Hospital, São Paulo, Brazil

**Keywords:** HLA, Supertypes, Human populations, Natural selection, Pathogens, Adaptation

## Abstract

Supertypes are groups of human leukocyte antigen (HLA) alleles which bind overlapping sets of peptides due to sharing specific residues at the anchor positions—the B and F pockets—of the peptide-binding region (PBR). HLA alleles within the same supertype are expected to be functionally similar, while those from different supertypes are expected to be functionally distinct, presenting different sets of peptides. In this study, we applied the supertype classification to the HLA-A and HLA-B data of 55 worldwide populations in order to investigate the effect of natural selection on supertype rather than allelic variation at these loci. We compared the nucleotide diversity of the B and F pockets with that of the other PBR regions through a resampling procedure and compared the patterns of within-population heterozygosity (*He*) and between-population differentiation (*G*_ST_) observed when using the supertype definition to those estimated when using randomized groups of alleles. At HLA-A, low levels of variation are observed at B and F pockets and randomized *He* and *G*_ST_ do not differ from the observed data. By contrast, HLA-B concentrates most of the differences between supertypes, the B pocket showing a particularly high level of variation. Moreover, at HLA-B, the reassignment of alleles into random groups does not reproduce the patterns of population differentiation observed with supertypes. We thus conclude that differently from HLA-A, for which supertype and allelic variation show similar patterns of nucleotide diversity within and between populations, HLA-B has likely evolved through specific adaptations of its B pocket to local pathogens.

**Electronic supplementary material:**

The online version of this article (doi:10.1007/s00251-015-0875-9) contains supplementary material, which is available to authorized users.

## Introduction

The three classical human leukocyte antigen (HLA) class I genes, HLA-A, HLA-B, and HLA-C, are extremely polymorphic and exhibit thousands of alleles, most of them coding for different proteins (2112, 2789, and 1799 HLA-A, HLA-B, and HLA-C proteins currently defined, respectively) (Robinson et al. 2015). These molecules play a central role in the immune response by presenting processed peptides derived from proteins of the intracellular environment (including foreign ones derived from intracellular parasites such as viruses and some bacteria) to cytotoxic T lymphocytes and also functioning as ligands for the killer immunoglobulin-like receptor (*KIR*) of natural killer cells (Parham 2005).

Almost all of the HLA class I polymorphisms are clustered in exons 2 and 3, which code for the α1 and α2 extracellular domains of the HLA molecule. These domains form a groove-like structure known as the peptide-binding region (PBR) which engages the peptides (Saper et al. 1991). At the DNA level, the PBR codons exhibit striking features regarding their diversity, including a high heterozygosity (Parham et al. 1989; Lawlor et al. 1990; Hedrick et al. 1991) and high rates of non-synonymous substitutions (Hughes and Nei 1988; Takahata et al. 1992). These characteristics contrast with neutral expectations and support the hypothesis that balancing selection has maintained variation at these codons. The high levels of variation observed at the sites involved in peptide binding support a model of host-pathogen coevolution (Apanius et al. 1997), which states that the pathogenic microorganisms are the main evolutionary force shaping HLA variation (Borghans et al. 2004; Slade and McCallum 1992; Takahata and Nei 1990). Further supporting this hypothesis, several studies have demonstrated a positive correlation between the diversity level of some HLA genes and the richness of environmental pathogens (Prugnolle et al. 2005; Qutob et al. 2011; Sanchez-Mazas et al. 2012). These results corroborate the idea that the codons making up the PBR constitute the main targets of balancing selection within HLA genes. However, the analyses performed to date generally treat the PBR region as a homogeneous block, whereas it is in fact composed of six different pocket-like structures (A, B, C, D, E, and F). Each pocket accommodates one of the nine amino acid residues of the bound peptide (the first, second, third, sixth, seventh, and ninth, respectively) (Saper et al. 1991). Moreover, the binding affinity between a given HLA molecule and a specific peptide depends on the chemical properties of each PBR pocket (Saper et al. 1991).

The strongest interaction between HLA molecules and the bound peptides is accounted by the B and F pockets, which accommodate the second and ninth amino acid residues of the peptide, respectively (Saper et al. 1991). As the amino acids composing the B and F pockets play a central role in peptide recognition by the HLA molecules, Sidney et al. (1996) classified HLA alleles into *supertypes*, defined as groups of alleles sharing chemical properties at the B and F pockets. The logic behind the classification is that alleles within supertypes are expected to exhibit widely overlapping peptide repertoires, whereas alleles from different supertypes would more frequently bind non-overlapping sets of peptides. Supertypes were originally defined by sequencing endogenously bound ligands and searching for motifs shared by alleles that bind similar peptides and by analyzing the three-dimensional structure of the HLA molecules (Sette and Sidney 1999; Sidney et al. 1996, 2008). As a result, four supertypes were described for HLA-A (A1, A2, A3, and A24) and five for HLA-B (B7, B27, B44, B58, and B62), and they were originally assigned, respectively, to 31 HLA-A and 57 HLA-B alleles whose peptide-binding specificities were experimentally defined. These alleles were used to construct a reference panel for the B and F amino acid sequences. A set of 945 HLA-A and HLA-B alleles with unknown binding specificities were then checked for matches to the sequences of this panel (Sidney et al. 2008). Among these 945 previously unclassified alleles, 57 % presented a full match in both B and F pockets to alleles with known supertype status. Another 23.8 % presented partial matches with residues found in these pockets.

In line with the expectation that supertypes constitute a functionally relevant definition of HLA variation, several researchers have found that grouping alleles into supertypes is useful in disease association studies involving HLA loci (Alencar et al. 2013; Chakraborty et al. 2013; Cordery et al. 2012; Gilchuk et al. 2013; Karlsson et al. 2012, 2013; Kuniholm et al. 2013; Trachtenberg et al. 2003), allowing large numbers of rare alleles to be grouped according to a functional criterion, thus increasing the power of the studies. From an evolutionary point of view, natural selection is expected to leave a detectable signature on B and F pockets and, consequently, on the genotypes defined by examining HLA variation from the perspective of supertypes. For example, under the assumption that pathogen-driven selection shapes supertype frequencies, we expect genetic variation defined at the supertype level to show patterns of polymorphism and differentiation indicative of balancing selection to a greater degree than variation that is not related to supertype definition. The prediction that balancing selection on supertype variation would result in detectable genetic signatures was raised by Sette and Sidney (1999), who found that “supertype frequencies were high and fairly conserved among different ethnicities.” In addition, Naugler and Liwski (2008) argued that “natural selection should favor maximization of the heterozygosity of allele supertypes instead of the heterozygosity of individual alleles,” making explicit the hypothesis that supertypes, as defined by B and F pocket variations, constitute the level of variation that is the primary target of natural selection in HLA genes.

Both conservation of supertype frequencies between populations and increased heterozygosity at the supertype level are expected to generate a pattern of low-population differentiation when compared with those observed at the allelic level. Balancing selection at the supertype level would also enhance genetic variation at the B and F pockets compared with other regions of the PBR, increasing the chances of antigen recognition by the immune system. However, testing these hypotheses, i.e., comparing population differentiation and variability defined at the levels of HLA alleles and supertypes, respectively, represents a methodological challenge due to the difficulties in comparing measures of differentiation and heterozygosity for genetic variants that are defined by different attributes (alleles being defined by all variation in the coding region, by contrast with supertypes which are defined by a subset of codons). Indeed, because supertypes are sets of alleles, genetic variation defined at the allele level is nested within that defined at the supertype level. Therefore, heterozygosity at the supertype level is constrained to be lower or equal to that estimated at the allele level. Furthermore, because population genetic differentiation measured by statistics related to Wright’s *F*_ST_ is strongly determined by intrapopulation variability (Jost 2008), we expect higher levels of population differentiation at the supertype level simply because of the decreased number of supertype variants in comparison to alleles.

In the present study, our aim is to investigate whether the use of supertype instead of allele definitions at HLA-A and HLA-B loci reduces population differentiation and increases heterozygosity, as expected under a model of balancing selection acting on supertypes. For the reasons explained above, we control our analyses for the inherent differences in polymorphism between these two kinds of classification. Our approach consists in producing null distributions for population differentiation and heterozygosity by generating randomized sets of alleles (herein referred to as “random supertypes”) that match true supertype sampling properties (i.e., number of supertypes and number of alleles per supertype) without any biological criteria for pooling them together. We also analyze supertype variation at the nucleotide level by partitioning DNA sequences into segments corresponding to the different pockets within the PBR. Our hypothesis is that the B and F pockets, which are the major determinants of the peptide-binding specificities and used to define supertypes, constitute the main targets of balancing selection and thus retain higher levels of diversity compared to other PBR pockets.

## Materials and methods

### Population data

We used a database generated for the 13th International Histocompatibility Workshop (IHWS) (Mack et al. 2006) from which we excluded populations presenting (a) an allelic resolution lower than the first two sets of digits (now referred to as second field level of resolution), so as to only keep alleles differing at the protein level; (b) genotypic ambiguities; and (c) deviation from Hardy-Weinberg expectations. This filtering resulted in a dataset of 6435 and 6409 individuals typed for HLA-A and HLA-B, respectively, belonging to 55 different populations: seven sub-Saharan African (SSA), two North African (NAF), eight Southwest Asian (SWA), four European (EUR), 22 Southeast Asian (SEA), four Pacific islanders (PAC), four Australian aborigine (AUS), two North Asian (NEA), and two Native American (AME) populations (Supplementary Material Table 1-S). Almost half of these populations (24 out of 55) had demographic histories indicating that they were likely to have experienced severe founder effects (these populations were from Oceania, Taiwan, and the Americas). Because such reductions in diversity due to demographic effects can potentially mask signals of balancing selection, we carried out all the analyses with both the complete set of 55 populations and a reduced set of 30 populations (obtained by excluding those from Oceania, Taiwan, and the Americas).

### Supertype definition

**Fig. 1:**
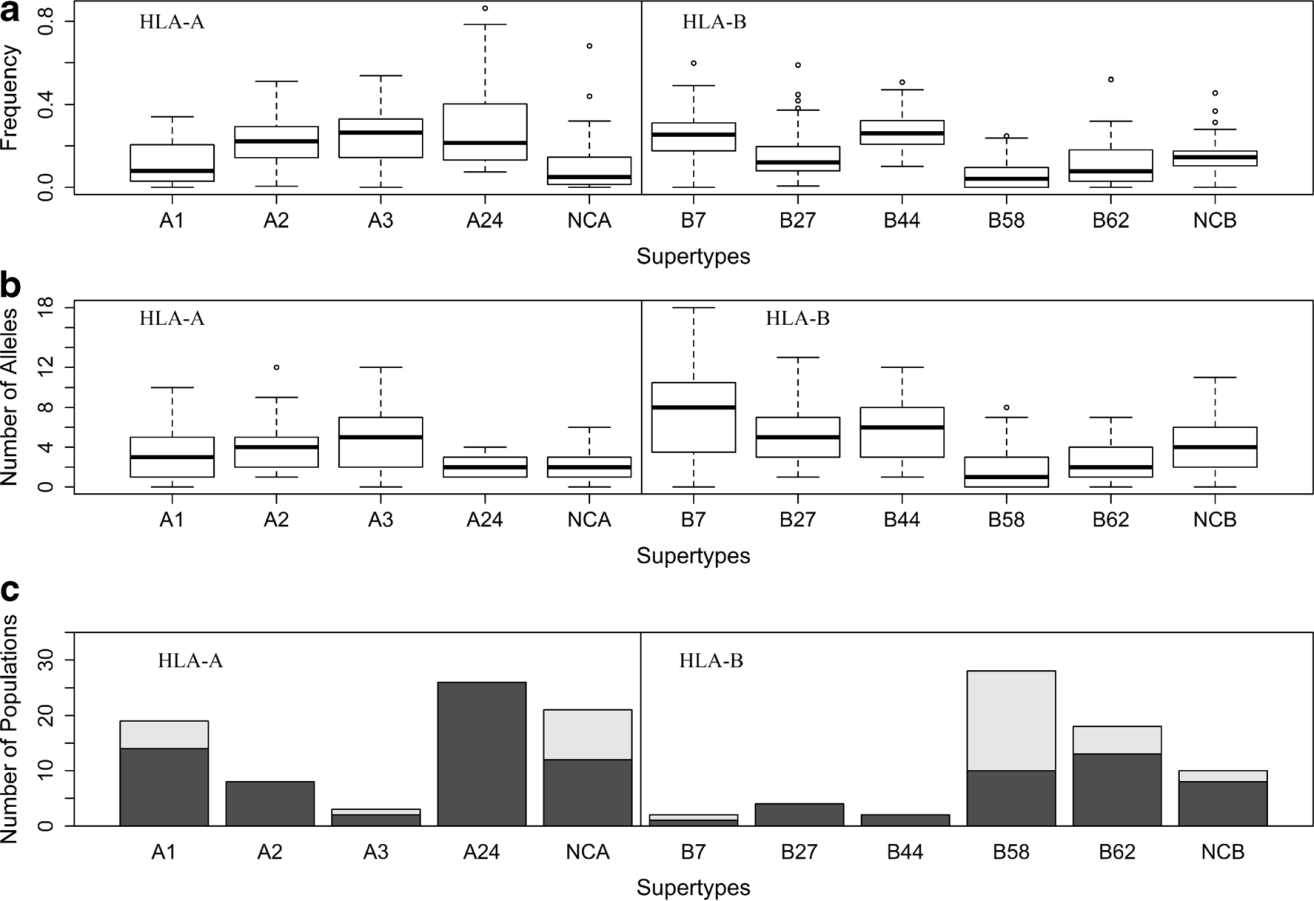
Supertype variation, **a** *boxes* represent the frequency distributions of the four HLA-A and the five HLA-B supertypes and the “non-classified alleles” NCA and NCB, respectively; **b** *each box* represents the distribution of the number of distinct alleles of each supertype per population; and **c** the *dark gray section of the bars* represents the number of populations showing only one allele for the referred supertype (referred to as “monomorphic populations”). The *light gray section of the bars* represents the number of populations where the referred supertype was not detected

We assigned all *HLA*-*A* and *HLA*-*B* alleles to their specific supertype as defined by the classification given in figures 1 (http://www.biomedcentral.com/1471-2172/9/1/figure/F1) and 2 (http://www.biomedcentral.com/1471-2172/9/1/figure/F2) from Sidney et al. (2008). The alleles not assigned to any supertype were treated in our analyses of population differentiation and molecular variation in two ways: (a) their allele-level definition was used and (b) they were pooled into groups of “non-classified alleles” (named NCA and NCB for HLA-A and HLA-B, respectively). We included A*29:01, A*29:02, A*29:03, A*30:01, A*30:08, and A*68:06 in the NCA group because of their ambiguous supertype allocation (Sidney et al. 2008), and all B*08 alleles were assigned to the NCB group because of their unique PBR structures, which make the peptide-binding profile unpredictable (Sidney et al. 2008).

### Population genetic analyses

We tested the population samples for deviation from Hardy-Weinberg (HW) equilibrium using the Gene[rate] program which tests the null hypothesis of equilibrium on the basis of a log-likelihood ratio test on frequency estimates (both under HW and under a generalized non-HW model) (Nunes et al. 2014; Nunes 2014).

We wrote R scripts to estimate supertype frequencies by direct counting of alleles, generate summary statistics (number of alleles (k) and expected sample heterozygosity (*He*)), and estimate genetic differentiation between pairs of populations by using *G*_ST_ (Nei and Chesser 1983). Mantel tests (Mantel 1967) for assessing Pearson’s correlations between genetic distances obtained either from supertype or from allelic data were carried out using the ade4 R package (Dray and Dufour 2007), and all graphs and other statistical tests (e.g., Wilcoxon rank sum test) were also generated using R version 3.0.2 (Development Core Team 2011). In box plots, the boxes correspond to the interquartile range, the median is the thick line inside the box, and whiskers extend up to observations that are outside the box for less than 1.5 times the interquartile range. Dots are outliers to these limits. By using Arlequin 3.5 program (Excoffier and Lischer 2010), we performed a hierarchical analysis of molecular variance (AMOVA) for each supertype taken individually by pooling all others into a unique group of “non-classified alleles” for the calculations. In this way, we estimated the diversity among populations (*F*_ST_), among populations within geographic regions (*F*_SC_), and among geographic regions (*F*_CT_) for each supertype.

#### Testing the molecular variation of the PBR pockets

We analyzed the molecular variation at each PBR pocket using the coding sequences of the six pockets which make up the HLA class I peptide-binding region (A to F). The definition of these codons (Table 1) was taken from Saper et al. (1991). The residues retained for the analysis of pocket B variability are the ones surrounding the rim and constituting the inner wall of the pocket. As the main-chain atoms of pocket B residues 24, 25, and 34 are part of the protein backbone, and their side chains are not turned to the pocket area, they are not expected to contribute to the chemical properties of the pocket, and were not included in the analysis (Saper et al. 1991; see also Table 1).

**Table 1:** Codon composition of the PBR pockets

Pockets	Codons	Total size in base pairs (bp)
A	5, 7, 59, 63, 66, 99, 159, 163, 167, and 171	30
B	7, 9, 24, 25, 34, 45, 63, 66, 67, 70, and 99	33
C, D, and E	9, 70, 73, 74, 97, 99, 114, 147, 152, 155, 156, 159, and 160	39
F	77, 80, 81, 84, 116, 123, 143, 146, and 147	27

From: Saper et al. (1991)

The B and F pockets were analyzed individually because of their central role in engaging peptides and in defining supertypes. As the C, D, and E pockets jointly make up the central region of the PBR and are shorter compared to other pockets, we pooled them for the present analysis. The A pocket was analyzed individually because of its position at one end of the PBR.

We estimated the nucleotide diversity (*π*) (Nei 1987) per pocket (i.e., A, B, pooled CDE, and F) for each population (referred to as *π*_total_). For these four pockets, we also computed within- and between-supertype nucleotide diversity (referred to as *π*_within_ and *π*_st_, respectively), and thus estimated a measure of among-supertype variation for each pocket, obtained using the following formula:

1 $$ {\pi}_{\mathrm{st}}=\frac{\pi_{\mathrm{total}}-{\pi}_{\mathrm{within}}}{\pi_{\mathrm{total}\;}} $$

Total, within- and between-supertype *π* values were calculated in two ways: (a) by excluding the non-classified alleles and (b) by including the non-classified alleles as a single group. As the dataset is limited to alleles defined at second field level of resolution, no information about synonymous polymorphism is available. We addressed this problem by applying the same strategy as described by Buhler and Sanchez-Mazas (2011), which consisted in treating as missing data the nucleotide positions which were described as synonymous (Robinson et al. 2015). We excluded sites having more than 5 % missing data.

#### Testing genetic differentiation between populations based on supertypes

To test whether the levels of genetic differentiation between populations differed from those expected under the null hypothesis that supertypes are equivalent to random sets of alleles, we randomized the assignment of alleles into supertypes and calculated corresponding *He* and *G*_ST_ values. The randomized assignment of alleles to supertypes was performed using two different approaches (for both the complete and the reduced datasets):

1. By fixing the number of alleles per supertype to that observed in the original dataset
2. Without any constraint on the number of alleles associated to a specific supertype

The randomizations were repeated 10,000 times, and *p* values were estimated empirically by determining the number of randomized datasets with *G*_ST_ values lower or *He* values higher than those observed for the true data.

## Results and discussion

### HLA-A and HLA-B supertype frequencies and their geographic distributions

**Fig. 2:**
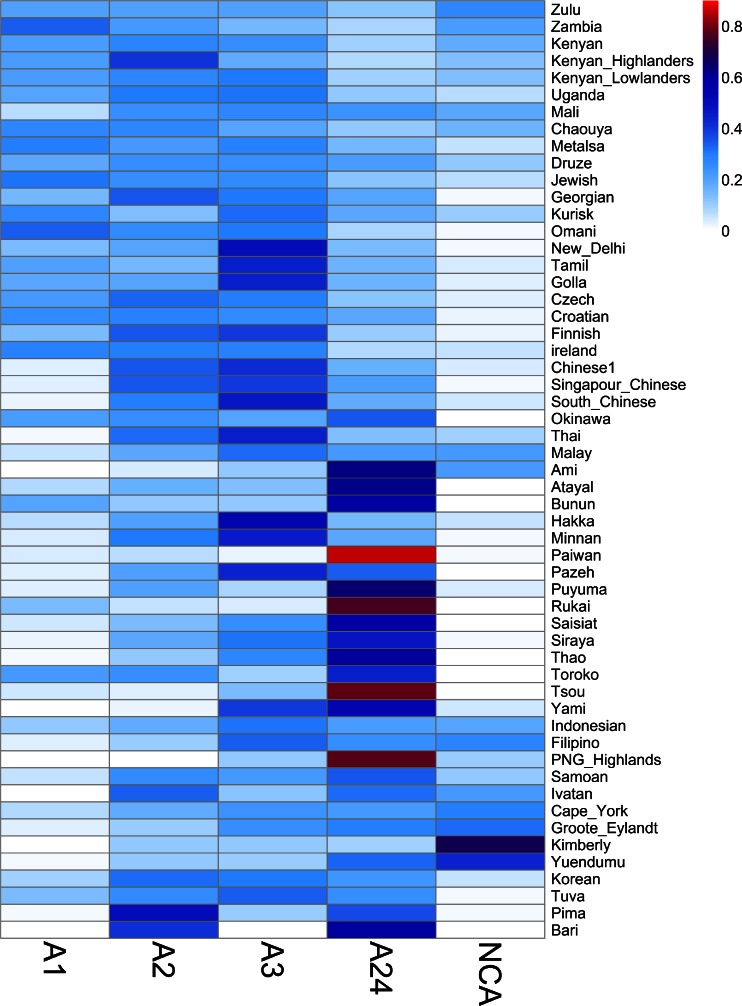
HLA-A supertype frequencies. Heat map summarizing the frequencies of the four *HLA*-*A* supertypes and the non-classified alleles (NCAs). Population names are shown on the *right*

**Fig. 3:**
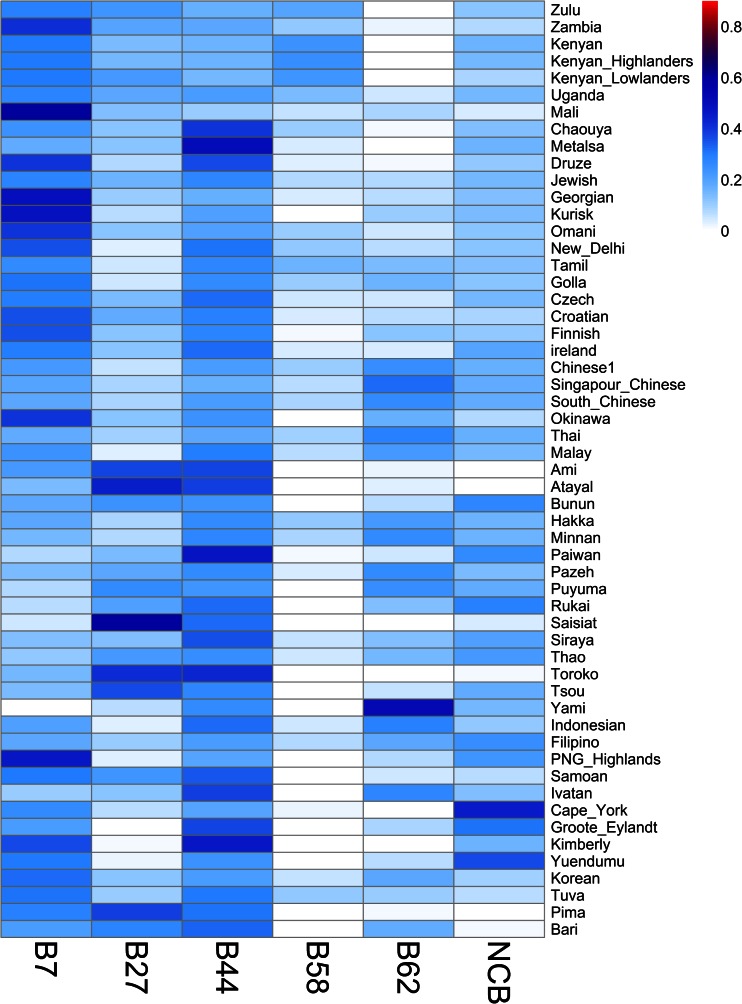
HLA-B supertype frequencies. Heat map summarizing the frequencies of the five *HLA*-*B* supertypes and the non-classified alleles (NCBs). Population names are shown on the *right*

In a previous study (the only one, to our knowledge, except our own study on HLA-DRB1 (Gibert and Sanchez-Mazas 2003)) addressing population differentiation at the supertype level, Sidney et al. (1996) used five population samples and reported that all supertypes were present in all world regions. This current study with 55 populations greatly extends those original observations, allowing us to show that some supertypes are not observed in all populations while reaching a frequency of more than 50 % in others (Figs. 1a, c, 2, and 3). Among the HLA-A supertypes, A1 is the rarest, showing frequencies smaller than 9 % in more than half of the populations (Fig. 1a) and being virtually absent in five of them (Fig. 1c). A1 alleles are found with high frequencies (22 % in average) in Africa, Southwest Asia, and Europe (Fig. 2), resulting in a significant geographic structure, i.e., with most of the variation being found among populations of different geographic regions (*F*_CT_ > *F*_SC_; Table 2). The A1 supertype is represented by a small number of alleles, with one or two alleles in more than half of the populations (Fig. 1b) and only one in 14 of them (Fig. 1c). The A2 and A3 supertypes exhibit more even distributions, half of the populations having frequencies ranging from 14 to 29 % for A2 and 14 to 32 % for A3 (Figs. 1a and 2). As a consequence, among the HLA-A supertypes, A2 and A3 present either the lowest or no geographic structure at all (*F*_CT_ < *F*_SC_ for A2 and *F*_CT_ not significantly different from 0 for A3; Table 2). All populations present at least one allele of supertype A2 (eight of them showing just one), while the A3 supertype is represented by a large number of alleles (Fig. 1b, c). The A24 supertype is observed in all populations (Fig. 1c), with frequencies ranging from 13 to 40 % in half of them (Fig. 1a). Despite its broad distribution, A24 is often represented by only two alleles, A*23:01 and A*24:02, with 26 and 10 populations showing just one or both of these alleles, respectively (Fig. 1b, c). This supertype is found at higher frequencies (40 % in average) in SEA, PAC, AUS, NEA, and AME (Fig. 2). Although A24 exhibits the highest level of population differentiation among the four HLA-A supertypes (*F*_ST_ = 11 %, *p* < 0.0001), most of the variation is found within geographic regions (*F*_CT_ < *F*_SC_). The frequencies of the HLA-A non-classified alleles (NCAs) vary greatly between populations, ranging from 2 to 14 % in half of them (Fig. 1a). The NCA group presents a strong geographic structure (*F*_CT_ being twice as much as *F*_SC_) and a very high *F*_ST_ value (almost 16 %) (Table 2). The highest NCA frequencies are found in African and Australian populations (averages of 16 and 43 %, respectively) (Fig. 2).

**Table 2:** Supertype differentiation indexes among populations (*F*_ST_), among populations within geographic regions (*F*_SC_), and among geographic regions (*F*_CT_)

Supertypes	*F*_ST_	*F*_SC_	*F*_CT_^a^
A1	9.95 %***	2.67 %***	7.48 %***
A2	4.85 %***	3.40 %***	1.51 %*
A3	6.48 %***	6.48 %***	0.000^b^
A24	11.14 %***	6.66 %***	4.80 %***
NCA	15.90 %***	4.90 %***	11.56 %***
B7	5.11 %***	3.21 %***	1.97 %*
B27	7.54 %***	7.10 %***	0.47%^b^
B44	3.21 %***	1.72 %***	1.51 %**
B58	8.34 %***	2.91 %***	5.59 %**
B62	11.38 %***	7.35 %***	4.35 %*
NCB	7.02 %***	2.90 %***	4.24 %***

*p < 0.01; **p < 0.001; ***p < 0.0001, where *p* values refer to the probability of observing a statistic as extreme under the null hypothesis of no structure

^a^In italics: Values of *F*_CT_ > *F*_SC_, an indication that most of the variation was found among populations of different geographic regions

^b^Not significant value

The HLA-B supertypes fall into two main categories regarding their frequency distributions. On the one hand, B7 and B44 exhibit a pattern resembling A2 and A3, with high average frequencies (Figs. 1a and 3) and relatively low levels of geographic structure (Table 2). Half of the populations present frequencies ranging from 18 to 31 % for B7 and from 21 to 32 % for B44, respectively (Figs. 1a and 3). Both B7 and B44 are observed in all populations (except B7 in the Yami; Figs. 1c and 3), with large numbers of alleles per population (Fig. 1b, c). By contrast, B58 and B62 exhibit very low frequencies, ranging from 0 to 5.8 % and from 2.9 to 18 % in half of the populations, respectively (Fig. 1a). Among the five HLA-B supertypes, B62 presents the highest level of population differentiation (*F*_ST_ = 11.38 %, *p* < 0.0001; Table 2), although with no clear geographic structure (*F*_CT_ < *F*_SC_; Table 2). Such a geographic structure is only found for B58 (*F*_CT_ of 5.6 %, almost twice as great as *F*_SC_; Table 2), which is observed in SSA populations at an average frequency of 33 % (from 23 to 60 %; Fig. 3), against 4.2 % in the other regions (Fig. 3) and no observation at all in many populations (18 out of 55; Fig. 3). The B27 supertype presents an intermediate pattern between B7/B44 and B58/B62. It exhibits relatively lower frequencies (from 7 to 19 % in half of the populations; Fig. 1a) and a higher level of population differentiation than B7 and B44 (*F*_ST_ = 7.5 %, *p* < 0.0001; Table 2) but no geographic structure (*F*_CT_ very close to zero; Table 2). Contrasting with what is observed for the NCA, the non-classified alleles for HLA-B (NCB) are quite frequent, with frequencies ranging from 10 to 17 % in half of the populations (Fig. 1a). More than 75 % of populations present at least two different NCBs (Fig. 1b), and only two populations lack one of these alleles (Fig. 1c). The NCBs also exhibit a significant geographic structure, although not as strong as for NCA (Table 2).

In summary, based on the observed data, supertypes can be allocated into two main categories: on the one hand, A2, A3, B7, B27, and B44 fit the classical view that supertypes are evenly distributed (Figs. 1a, 2, and 3), poorly structured geographically (Table 2), and represented by a large number of alleles (Fig. 1b, c). On the other hand, A1, A24, B58, and B62 present a greater frequency variation among populations (Figs. 2 and 3 and Table 2), and in some cases significant geographic structure (i.e., for A1 and B58, both being very common in Africa), and are represented by a smaller number of alleles. Although the unclassified alleles have brought noise to the analysis, they should not be ignored. They are a consequence of the functional supertype classification, and they were kept to understand exactly how they influence the variations in HLA-A and HLA-B. As discussed above, the NCA consists of a small group of alleles, which reach high frequencies in island populations. On the other hand, NCB is a more heterogeneous group appearing in almost all populations.

### Heterozygosity and interpopulation differentiation

Using both complete and reduced datasets (see “Materials and methods” section), the heterozygosity estimated for the data treated at the allelic level is always larger than that estimated for the data treated at the supertype level (Table 3). This result is expected because alleles are nested within supertypes, and the heterozygosity of the latter is thus constrained to be equal to or smaller than that of the former.

**Table 3:** Expected heterozygosity (*He*) of alleles and supertypes

Loci	Dataset^a^	Average allelic *He*	Average supertype *He*
HLA-A	Complete	0.7761	0.6774
HLA-A	Reduced	0.8974	0.7504
HLA-B	Complete	0.8948	0.7577
HLA-B	Reduced	0.9429	0.7766

^a^Complete dataset, all populations; reduced dataset, excluding Pacific, Australian, Taiwanese, and Native American populations

**Fig. 4:**
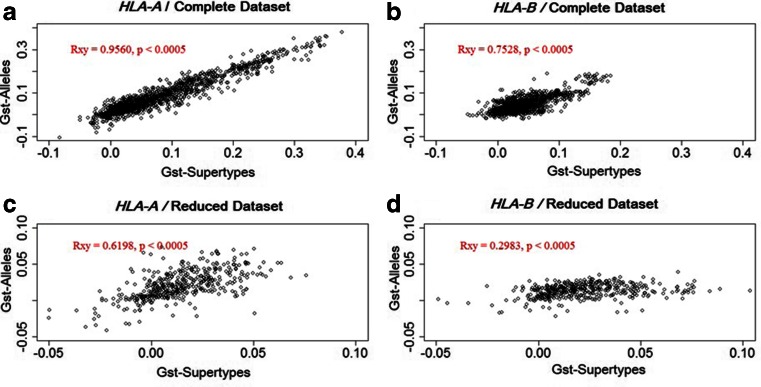
Plots of *G*_ST_ values between populations based on allele (*Y* axis) and supertype (*X* axis) frequencies. The correlation (*Rxy*) and significance were obtained using a Mantel test. Complete dataset, all populations and reduced dataset, excluding Pacific, Australian, Taiwanese, and Native American populations

In order to define the degree to which genetic differentiation, measured by *G*_ST_ between populations, was concordant at the supertype and allelic levels, we estimated the correlation between these measures and tested their significance using Mantel tests. The results suggest that when using the complete population dataset, the patterns of population differentiation observed at the supertype and allelic levels are very similar, especially for HLA-A (*r* = 0.956, *p* < 0.0005; Fig. 4a) but also for HLA-B (*r* = 0.75, *p* < 0.0005; Fig. 4b). The removal of the Pacific, Australian, Taiwanese, and Native American populations provokes an overall drop of both the *G*_ST_ values and their correlations. Despite this decrease, a high-correlation coefficient is still observed for HLA-A (*r* = 0.62, *p* < 0.0005; Fig. 4c), whereas the value is much lower for HLA-B (*r* = 0.3, *p* < 0.0005; Fig. 4d). Because Pacific, Australian, Taiwanese, and Native American populations contribute to large differentiation values, lower-correlation coefficients were expected after removing them. Furthermore, these populations also exhibit a reduced set of alleles per supertype, which may explain the higher correlations between alleles and supertypes when they are taken into account. The difference between alleles and supertypes is less pronounced for HLA-A which presents a smaller number of alleles per supertype in all populations (Fig. 1b, c).

### Patterns of molecular variability for different PBR pockets of HLA-A and HLA-B

Our goal in this part of the study was to test the prediction that the B and F pockets of the PBR exhibit the highest levels of variation as a consequence of their crucial role in peptide binding, which is expected to result in a stronger effect of balancing selection.

**Fig. 5:**
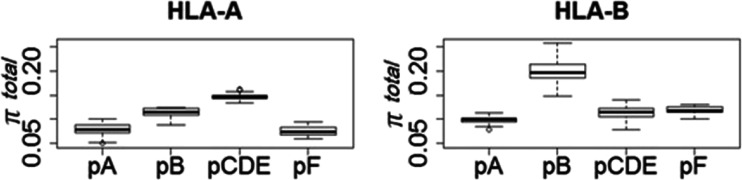
Total nucleotide diversity (*π*_total_) at HLA-A and HLA-B PBR pockets. *Each box* represents the distribution of the total nucleotide diversity per pocket for the populations of the complete dataset

We first estimated the global levels of variation at the PBR and observed significantly higher levels of nucleotide diversity (*π*_total_) at HLA-B, compared to HLA-A (*p* < 0.0000005; Wilcoxon rank sum test). Moreover, these two genes differ in the way molecular variation is distributed among the A, B, CDE, and F pockets within the PBR (Fig. 5). The rank order of *π*_total_ is *p*CDE ≫ *p*B ≫ *p*A > *p*F, at HLA-A, and *p*B ≫ *p*F > *p*CDE ≫ *p*A, at HLA-B (where *p* is an abbreviation for “pocket” and ≫ and > indicate greater than and significant, at the 0.00001 level, and greater than but non-significant differences, respectively, according to a Wilcoxon rank sum test; Fig. 5). Among the HLA-A pockets, most of the variation is found in the CDE pockets, which makes up the central region of the PBR, and significantly less in *p*B (*π*_total_ values ranging from 0.14 to 0.15 and from 0.11 to 012 in half of the populations, respectively; Fig. 5). The *p*A and *p*F pockets exhibit the smallest levels of variation (*π*_total_ values ranging from 0.07 to 0.09 in half of the populations; Fig. 5). Among the HLA-B pockets, *p*B exhibits by far the highest variation, with *π*_total_ values ranging from 0.18 to 0.21 in half of the populations, whereas the other pockets exhibit a relatively narrow *π*_total_ distribution (ranging from 0.10 to 012 in half of the populations; Fig. 5).

The hypothesis that the pockets B and F are the main targets of balancing selection is thus partially supported for HLA-B, since *p*B presents by far the highest level of nucleotide diversity. Interestingly, van Deutekom and Kesmir (2015) recently showed that changes involving several of the B pocket’s amino acids had a profound impact on peptide-binding properties, which corroborates our interpretation. On the other hand, *p*F, which is not significantly different from *p*A at HLA-A, and from *p*CDE at HLA-B, does not present an increased value of *π*_total_ which would be an evidence against balancing selection. It is important to note that these results were obtained independently from the classification of alleles into supertypes, since the determination of the pockets’ codons was taken from the classical study of Saper et al. (1991).

**Fig. 6:**
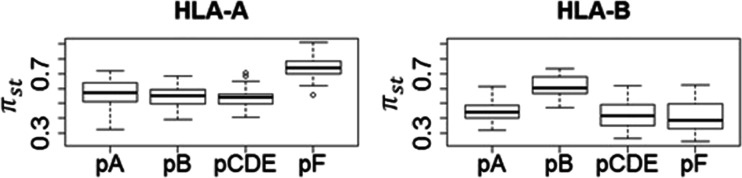
Nucleotide diversity between supertypes (*π*_st_) at HLA-A and HLA-B PBR pockets. *Each box* represents the distribution of the nucleotide diversity between supertypes per pocket for the populations of the complete dataset

We also analyzed how the nucleotide diversity was distributed between supertypes. Since the supertype categorization is based on variations of *p*B and *p*F, these pockets were expected to present more differences between supertypes than the others. This prediction was confirmed for *p*F at HLA-A and *p*B at HLA-B (Fig. 6).

As *p*B presents the highest levels of variation at HLA-B and also accounts for most of the differences between HLA-B supertypes, we conclude that the variation between HLA-B supertypes accounts for most of the differences observed between HLA-B alleles. In other words, alleles classified within a same HLA-B supertype share more similarities than alleles assigned to different HLA-B supertypes. By contrast, most of the differences between HLA-A supertypes lie within *p*F, the pocket presenting the lowest *π*_total_ values for this gene. Therefore, at this locus, the supertypes do not account for most of the variation between alleles (Fig. 6). In other words, HLA-A presents more variation within than between supertypes.

### Simulation approach to test selection on supertypes

According to the definition of Sidney et al. (1996), alleles included within the same supertype have overlapping peptide-binding specificities. To test the effects of the supertype classification on expected heterozygosities (*He*) and pairwise differentiation (*G*_ST_), we generated null distributions for these two statistics under the hypothesis that alleles within supertypes are a random collection, with no shared functional attributes. To this end, the assignment of alleles to supertypes was randomized by permuting the supertype labels attributed to each allele motif, as described in the “Materials and methods” section. As the same patterns were obtained using the two different simulation approaches (see “Materials and methods” section), we only present the results for the case without any constraint on the number of alleles associated to a specific supertype.

**Fig. 7:**
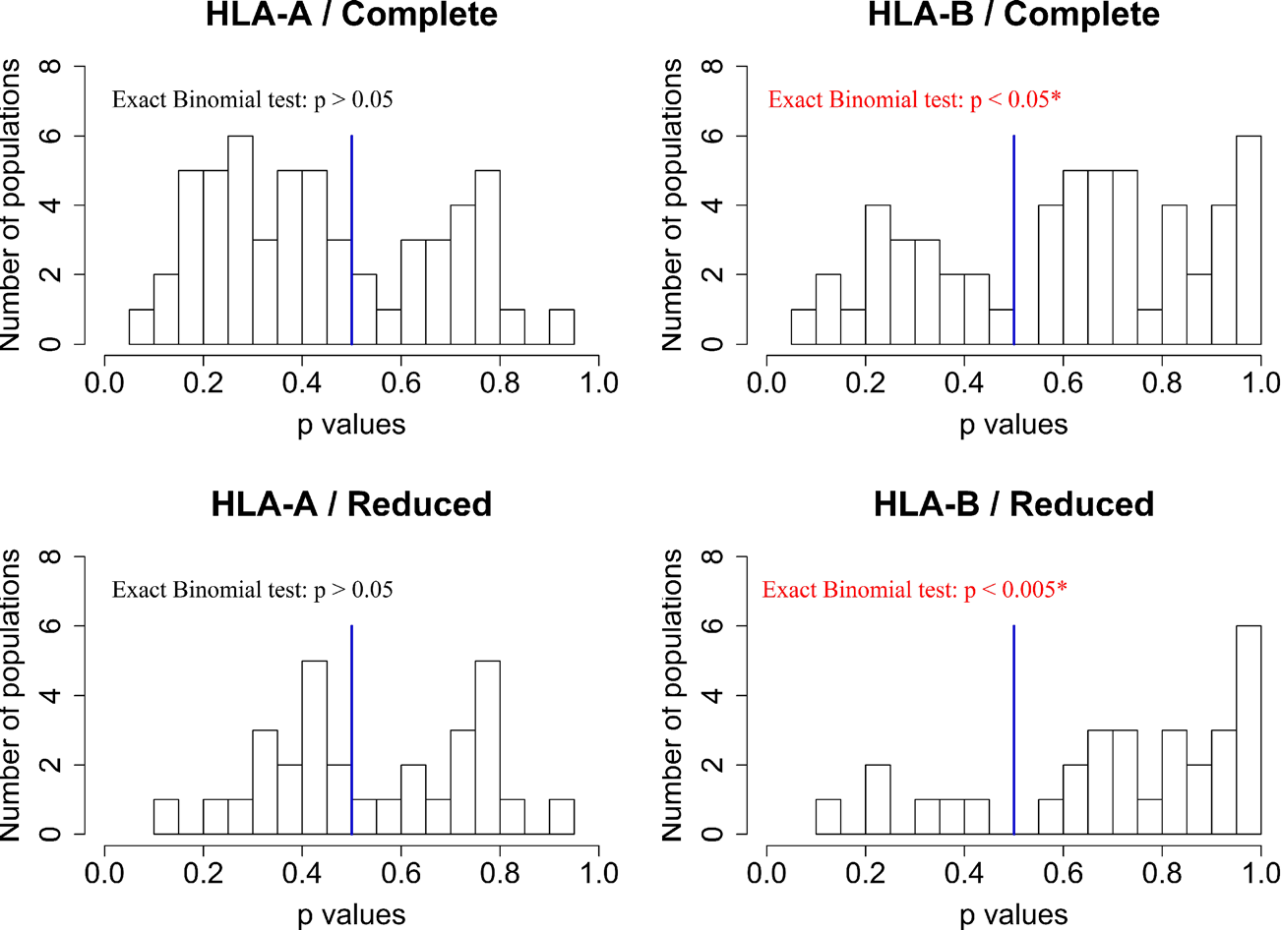
*P* value distributions obtained through simulations for the expected heterozygosity (*He*). The *p* value is defined as the proportion of simulated datasets with *He* larger than the observed *He*. The results obtained with the complete (*top*) and reduced (*bottom*) dataset are shown

For HLA-A, we do not observe any population with a significant difference in *He* in contrasts between the real and random supertype assignments. For HLA-B, 6 out of 55 populations exhibit significantly lower *He* (permutation-based *p* < 0.05) than those acquired via simulations. These six populations belong to the reduced dataset. Because the number of populations with individually significant *p* values in either direction (i.e., with significantly lower or greater *He* compared to the simulated value) is small, we investigated whether the distribution of the *p* values itself was informative regarding selective effects. To do this, we used an exact binomial test to assess whether the observed distribution of *p* values deviated from one composed of equal numbers of values on either side of 0.5 (the expected proportion of deviation in either direction under the null hypothesis; Fig. 7). For HLA-A, no significant deviation is found (*p* value > 0.05 for both complete and reduced datasets). For HLA-B, however, a significant skew towards *p* values greater than 0.5 is observed, indicating an overall significant excess of populations with lower *He* than those obtained through simulations (*p* value < 0.05 and *p* value < 0.005 for complete and reduced datasets, respectively).

**Fig. 8:**
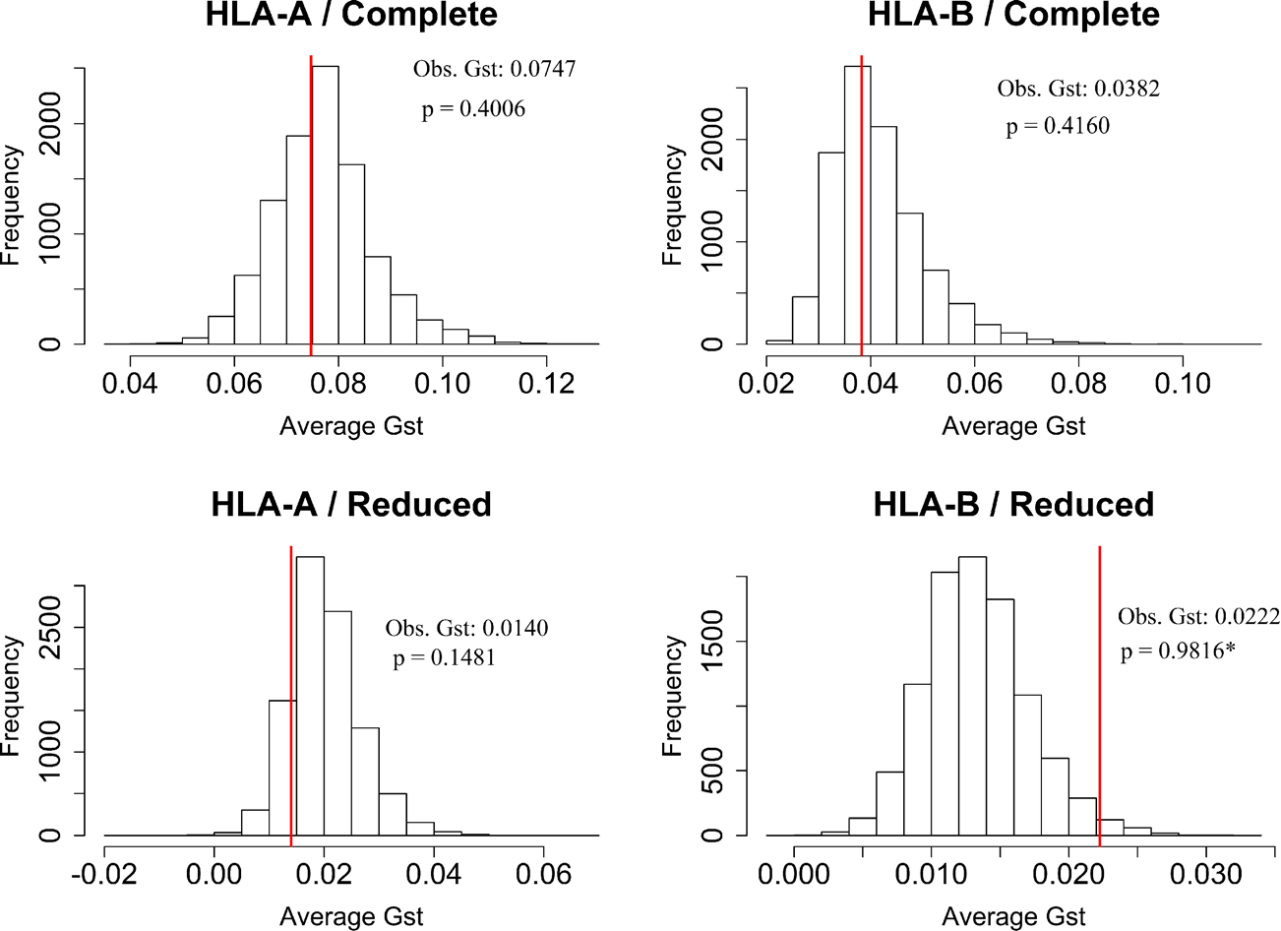
Simulation results for *G*_ST_. The *red line* represents the average observed *G*_ST_. We calculated the average *G*_ST_ value for each simulated step and then determined the significance as the proportion of simulated values smaller than the observed one. The results with the complete (*top*) and reduced (*bottom*) datasets are shown

For both HLA-A and HLA-B, *G*_ST_ values were not significantly different from those of the randomized data, when using the complete dataset. This is also true when using the reduced dataset for HLA-A but not for HLA-B. Indeed, after removing the Pacific, Australian, Taiwanese, and Native American populations, the observed *G*_ST_ is higher than 98 % of the simulations for HLA-B (Fig. 8). This finding differs from the expectations of Sidney et al. (1996), who predicted an overall decrease of differentiation at the supertype level. However, it is in agreement with our description of the observed data. Indeed, in our simulations, alleles were randomly assigned to supertypes, creating randomized supertypes with similar contents of common and rare alleles. The common alleles are expected to be assigned to different randomized supertypes in most of the simulations because they are less numerous than the rare alleles. Such a pattern is similar to that described for real HLA-A supertypes, which present a low number of common alleles per population (Fig. 1b, c). As discussed above, this pattern also explains the high correlation found between *G*_ST_ values measured at the allelic and supertype levels for this locus (Fig. 4). Finally, as also discussed above for the PBR pockets, less variation is found between than within HLA-A supertypes. This indicates that HLA-A supertypes are composed of heterogeneous sets of alleles with few sequence similarities at *p*F (Figs. 5 and 6), which explains the similarity between the results based on the observed and randomized data. On the other hand, HLA-B supertypes appear to be composed of alleles sharing more sequence similarities, as shown by the molecular analysis of the PBR pockets (Figs. 5 and 6).

In summary, HLA-B supertypes are sets of alleles with B pocket resemblances, and these similarities can be interpreted directly in terms of peptide presentation profiles because HLA-B supertypes exhibit major differences regarding the chemical properties of *p*B. Thus, our results showing an increased differentiation at the level of HLA-B supertypes are consistent with an effect of natural selection resulting in local adaptation of populations to different pathogen environments. Through our simulations, the functional grouping of alleles reflected by the HLA-B supertypes is disrupted, creating randomized groups in the same way as described for HLA-A. The frequent allocation of common alleles into different randomized supertypes in the simulations thus provokes both an increase of *He* and a decrease of population differentiations (*G*_ST_), when compared with the observed data (Figs. 7 and 8). In agreement with this interpretation, the inclusion of the Pacific, Australian, Taiwanese, and Native American populations reduces this effect because the patterns of variation at HLA-B for these populations resemble those observed at HLA-A, with a relatively low number of alleles belonging to different supertypes.

## Conclusions

The supertype classification of HLA-A and HLA-B alleles has been widely used in medical research, with reports suggesting that supertype-level variation explains susceptibility or resistance to a series of pathogenic diseases (Alencar et al. 2013; Chakraborty et al. 2013; Cordery et al. 2012; Gilchuk et al. 2013; Karlsson et al. 2012, 2013; Kuniholm et al. 2013; Trachtenberg et al. 2003). This classification was proposed in the 1990s as an attempt to find, as described by Sette and Sidney (1999), “the common denominators and similarities hidden within this very large degree of polymorphism.” The same authors also stated that “the overall frequency of each of these supertypes is remarkably high and fairly conserved among very different ethnicities. Thus, there might be some advantage for human populations to present approximately five to ten main binding specificities and that each one of these is maintained at relatively high frequency.” According to our results, the variation among HLA-B supertypes does reflect the functional diversity at this locus and is thus in agreement with the above-mentioned hypothesis. Our results strongly indicate that the B pocket is likely to be the main target of natural selection at HLA-B, as it presents the highest levels of molecular variation and accounts for the main differences in the peptide presentation profiles for this gene. However, in contrast with classical expectations for loci evolving under balancing selection, our simulation results reveal that HLA-B supertype frequencies do not show a signature of balancing selection (i.e., we find lower *He* compared to those of randomly assigned groups of alleles), implying that each supertype is not maintained at relatively high frequencies in all populations. This result is supported by the geographically heterogeneous distributions of B58 and B62 (and, to a lesser extent, B27) frequencies among populations. Moreover, populations are more differentiated than expected for HLA-B supertypes (higher observed *G*_ST_ values than those obtained from randomly assigned groups of alleles). As most of the differences between HLA-B supertypes lie in the B pocket, this means that the differences in HLA-B supertype composition among populations can be interpreted in terms of peptide recognition. Thus, for HLA-B, our results support the idea that populations present more differences in peptide presentation profiles than expected, possibly due to local adaptations to pathogens.

By contrast, most of the differences between HLA-A alleles are not related with differences at the supertype level. This is supported by our simulation results showing that the randomly assigned groups of alleles often reproduce the observed patterns of variation and differentiation of HLA-A supertypes. Moreover, HLA-A alleles are more conserved at the sites involved in peptide binding, suggesting that they present a more conserved profile of peptides across populations, differing from what is observed for HLA-B. Of note, one possible caveat of inferring peptide binding through the supertype classification is that some peptides presented by HLA class I molecules are known to assume a looping conformation outside the peptide-binding groove. However, no matter how different conformations a peptide can adopt, the anchor amino acids located at the peptide ends remain the same, limited by the B and F pockets. In this way, this conformational variability exhibited by the peptides is also a consequence of the interaction between the peptide anchors and the B and F pockets and thus is not expected to change the results obtained here.

Our results suggest that the B pocket of the HLA-B molecules is the main target of natural selection, whereas no such signals could be retrieved for the other HLA-B pockets nor for the pockets of the HLA-A molecules in relation to the supertype classification. This conclusion matches the expectations that supertypes are the primary targets of selection for HLA-B but not for HLA-A. Following this idea, we could state that HLA-A supertypes are composed by alleles whose resemblances are not the consequence of a shared phylogenetic origin. A future extension of this work could be to explore whether the central pockets C, D, and E that have been shown to contain most of the variation at HLA-A could be used as an alternate functional classification for these alleles.

## Electronic supplementary material

ESM 1 (PDF 230 kb)
