# Supplementary material for: HLA supertype variation across populations: new insights into the role of natural selection in the evolution of HLA-A and HLA-B polymorphisms
Source: Immunogenetics. 2015 Oct 12;67(11-12):651–63. doi: 10.1007/s00251-015-0875-9 (PMC4636516; doi:10.1007/s00251-015-0875-9)
Supplement: Supplementary file 1 — (PDF 230 kb) [file 251_2015_875_MOESM1_ESM.pdf]

Supplementary Material

Table 1-S. Samples sizes and geographic distribution.

Populations	Regions	N	
		HLA-A	*HLA-B*
[a]Zulu	SSA	186	201
[a]Zambia	SSA	43	44
[a]Kenyan	SSA	143	143
[a]Kenyan_Highlanders	SSA	241	240
[a]Kenyan_Lowlanders	SSA	265	265
[a]Uganda	SSA	163	161
[a]Mali	SSA	138	138
[a]Chaouya	NAF	67	68
[a]Metalsa	NAF	72	68
[a]Druze	SWA	100	100
[a]Jewish	SWA	117	109
[a]Georgian	SWA	105	107
[a]Kurisk	SWA	30	29
[a]Omani	SWA	119	120
[a]New_Delhi	SWA	66	66
[a]Tamil	SWA	50	49
[a]Golla	SWA	88	104
[a]Czech	EUR	105	106
[a]Croatian	EUR	150	150
[a]Finnish	EUR	90	90
[a]Ireland	EUR	999	1000
[a]Chinese1	SEA	149	149
[a]Singapour_Chinese	SEA	86	86
[a]South_Chinese	SEA	282	281
Okinawa	SEA	105	104
[a]Thai	SEA	98	99
[a]Malay	SEA	124	101
Ami	SEA	98	98
Atayal	SEA	106	106
Bunun	SEA	101	101
Hakka	SEA	55	55
Minnan	SEA	102	102
Paiwan	SEA	51	51
Pazeh	SEA	55	55
Puyuma	SEA	50	50
Rukai	SEA	50	50
Saisiat	SEA	51	51
Siraya	SEA	51	51
Thao	SEA	30	30
Toroko	SEA	55	55
Tsou	SEA	51	51
Yami	SEA	50	50
[a]Indonesian	SEA	50	49

[a]Filipino	SEA	94	94
PNG_Highlands	PAC	92	75
Samoan	PAC	50	50
Ivatan	PAC	50	50
Cape_York	AUS	103	100
Groote_Eylandt	AUS	75	75
Kimberly	AUS	36	38
Yuendumu	AUS	191	193
[a]Korean	NEA	191	200
[a]Tuva	NEA	188	180
Pima	AME	86	89
Bari	AME	92	82

[a]Populations included in the “Reduced Dataset”

Table 2-S. Supertypes frequencies

Populations	Region	HLA-A					HLA-B					
		A1	A2	A3	A24	NCA	B7	B27	B44	B58	B62	NCB
Zulu	SSA	0.201613	0.204301	0.206989	0.123656	0.263441	0.283582	0.233831	0.169154	0.189055	0.004975	0.119403
Zambia	SSA	0.337209	0.22093	0.151163	0.081395	0.209302	0.409091	0.193182	0.181818	0.113636	0.022727	0.079545
Kenyan	SSA	0.20979	0.276224	0.248252	0.090909	0.174825	0.297203	0.143357	0.157343	0.237762	0.003497	0.160839
Kenyan_Highlanders	SSA	0.215768	0.396266	0.172199	0.080913	0.134855	0.302083	0.145833	0.158333	0.247917	0	0.145833
Kenyan_Lowlanders	SSA	0.209434	0.266038	0.298113	0.09434	0.132075	0.30566	0.216981	0.15283	0.232075	0.003774	0.088679
Uganda	SSA	0.196319	0.303681	0.312883	0.116564	0.070552	0.273292	0.18323	0.21118	0.136646	0.049689	0.145963
Mali	SSA	0.068841	0.246377	0.264493	0.235507	0.184783	0.597826	0.126812	0.101449	0.054348	0.083333	0.036232
Chaouya	NAF	0.268657	0.268657	0.19403	0.11194	0.156716	0.235294	0.117647	0.397059	0.102941	0.014706	0.132353
Metalsa	NAF	0.291667	0.222222	0.284722	0.145833	0.055556	0.176471	0.117647	0.507353	0.036765	0.007353	0.154412
Druze	SWA	0.185	0.25	0.245	0.21	0.11	0.4	0.08	0.365	0.035	0.01	0.11
Jewish	SWA	0.307692	0.247863	0.25641	0.123932	0.064103	0.275229	0.16055	0.261468	0.077982	0.077982	0.146789
Georgian	SWA	0.147619	0.342857	0.3	0.195238	0.014286	0.490654	0.107477	0.168224	0.037383	0.065421	0.130841
Kurisk	SWA	0.266667	0.133333	0.316667	0.183333	0.1	0.482759	0.068966	0.206897	0	0.103448	0.137931
Omani	SWA	0.340336	0.252101	0.302521	0.088235	0.016807	0.404167	0.120833	0.2	0.104167	0.045833	0.125
New_Delhi	SWA	0.143939	0.19697	0.507576	0.136364	0.015152	0.356061	0.030303	0.310606	0.113636	0.068182	0.121212
Tamil	SWA	0.2	0.15	0.45	0.16	0.04	0.255102	0.05102	0.265306	0.153061	0.142857	0.132653
Golla	SWA	0.181818	0.193182	0.443182	0.153409	0.028409	0.307692	0.052885	0.259615	0.100962	0.158654	0.120192
Czech	EUR	0.22381	0.328571	0.290476	0.12381	0.033333	0.292453	0.141509	0.320755	0.051887	0.04717	0.146226
Croatian	EUR	0.253333	0.283333	0.253333	0.183333	0.026667	0.353333	0.176667	0.283333	0.036667	0.063333	0.086667
Finnish	EUR	0.138889	0.344444	0.394444	0.1	0.022222	0.355556	0.122222	0.272222	0.016667	0.122222	0.111111
Ireland	EUR	0.284785	0.295295	0.286286	0.074074	0.05956	0.2905	0.1195	0.317	0.0415	0.042	0.1895
Chinese1	SEA	0.030201	0.342282	0.422819	0.16443	0.040268	0.218121	0.057047	0.208054	0.104027	0.244966	0.167785
Singapour_Chinese	SEA	0.034884	0.343023	0.389535	0.215116	0.017442	0.19186	0.081395	0.168605	0.063953	0.319767	0.174419

South_Chinese	SEA	0.021277	0.29078	0.45922	0.179078	0.049645	0.181495	0.085409	0.213523	0.088968	0.252669	0.177936
Okinawa	SEA	0.214286	0.247619	0.190476	0.342857	0.004762	0.403846	0.120192	0.235577	0	0.163462	0.076923
Thai	SEA	0.010204	0.316327	0.44898	0.127551	0.096939	0.176768	0.090909	0.186869	0.090909	0.287879	0.166667
Malay	SEA	0.056452	0.181452	0.318548	0.221774	0.221774	0.237624	0.029703	0.292079	0.069307	0.222772	0.148515
Ami	SEA	0	0.040816	0.112245	0.627551	0.219388	0.22449	0.372449	0.377551	0	0.02551	0
Atayal	SEA	0.080189	0.169811	0.132075	0.617925	0	0.136792	0.448113	0.382076	0	0.033019	0
Bunun	SEA	0.193069	0.113861	0.108911	0.584158	0	0.188119	0.237624	0.237624	0	0.069307	0.267327
Hakka	SEA	0.063636	0.2	0.536364	0.145455	0.054545	0.181818	0.081818	0.254546	0.109091	0.218182	0.154545
Minnan	SEA	0.044118	0.29902	0.455882	0.186275	0.014706	0.147059	0.078431	0.264706	0.088235	0.259804	0.161765
Paiwan	SEA	0.039216	0.068627	0.019608	0.862745	0.009804	0.078431	0.137255	0.470588	0.009804	0.04902	0.254902
Pazeh	SEA	0.027273	0.2	0.436364	0.336364	0	0.136364	0.181818	0.254546	0.036364	0.254545	0.136364
Puyuma	SEA	0.03	0.2	0.09	0.64	0.04	0.08	0.26	0.23	0	0.25	0.18
Rukai	SEA	0.14	0.06	0.04	0.76	0	0.07	0.2	0.32	0	0.13	0.28
Saisiat	SEA	0.04902	0.137255	0.245098	0.568627	0	0.04902	0.588235	0.323529	0	0	0.039216
Siraya	SEA	0.019608	0.186275	0.313725	0.470588	0.009804	0.127451	0.127451	0.352941	0.058824	0.127451	0.205882
Thao	SEA	0.016667	0.116667	0.266667	0.6	0	0.116667	0.216667	0.25	0.05	0.15	0.216667
Toroko	SEA	0.218182	0.245455	0.090909	0.445455	0	0.145455	0.418182	0.427273	0	0	0.009091
Tsou	SEA	0.04902	0.029412	0.137255	0.784314	0	0.137255	0.362745	0.264706	0	0.058824	0.176471
Yami	SEA	0	0.02	0.39	0.54	0.05	0	0.07	0.26	0	0.52	0.15
Indonesian	SEA	0.11	0.18	0.31	0.21	0.19	0.204082	0.030612	0.316327	0.05102	0.285714	0.112245
Filipino	SEA	0.031915	0.106383	0.340426	0.244681	0.276596	0.180851	0.101064	0.207447	0.074468	0.18617	0.25
PNG_Highlands	PAC	0	0.005435	0.108696	0.782609	0.103261	0.46	0.033333	0.193333	0	0.08	0.233333
Samoan	PAC	0.06	0.26	0.22	0.35	0.11	0.3	0.23	0.35	0	0.05	0.07
Ivatan	PAC	0	0.34	0.12	0.32	0.22	0.1	0.12	0.38	0	0.27	0.13
Cape_York	AUS	0.072816	0.174757	0.237864	0.223301	0.291262	0.255	0.07	0.195	0.025	0	0.455
Groote_Eylandt	AUS	0.033333	0.106667	0.246667	0.293333	0.32	0.213333	0.006667	0.373333	0.006667	0.086667	0.313333
Kimberly	AUS	0	0.111111	0.111111	0.097222	0.680556	0.368421	0.013158	0.460526	0	0	0.157895
Yuendumu	AUS	0.010471	0.112565	0.10733	0.329843	0.439791	0.305699	0.020725	0.240933	0	0.064767	0.367876

Korean	NEA	0.094241	0.32199	0.301047	0.227749	0.054974	0.3175	0.125	0.215	0.06	0.185	0.0975
Tuva	NEA	0.140957	0.255319	0.340426	0.25	0.013298	0.313889	0.102778	0.3	0.108333	0.105556	0.069444
Pima	AME	0.011628	0.511628	0.104651	0.360465	0.011628	0.286517	0.382022	0.308989	0	0.016854	0.005618
Bari	AME	0.005435	0.407609	0	0.586957	0	0.207317	0.27439	0.329268	0	0.176829	0.012195
